# Spatiotemporal pulse weaving scalar optical hopfions

**DOI:** 10.1038/s41377-023-01101-w

**Published:** 2023-03-02

**Authors:** Chenhui Li, Sicong Wang, Xiangping Li

**Affiliations:** Guangdong Provincial Key Laboratory of Optical Fiber Sensing and Communications, Institute of Photonics Technology, Jinan University, Guangzhou, China

**Keywords:** Nanophotonics and plasmonics, Micro-optics

## Abstract

Scalar optical hopfions weaved by nested equiphase lines in the shape of a toroidal vortex are theoretically designed and experimentally demonstrated. This category of hopfions manifesting as a spatiotemporally structured pulse propagating in space-time may enable encoding and transferring optical topological information in an additional (temporal) dimension.

Topological solitons with topologically protected spin textures are cutting-edge researches in high-energy and condensed matter physics. Numerous studies have focused on low-dimensional magnetic topological solitons such as one-dimensional (1D) magnetic domain walls^1^ and 2D magnetic skyrmions^2^, and explicitly revealed the promising applications of topological solitons in spintronic devices. 3D topological textures with more complex features have also been attracting considerable interest in numerous branches of physics. For example, hopfions, as the most classic 3D topological solitons originally raised by Heinz Hopf in 1931^3^, have been proved to be of great significance in chiral magnets, quantum fields, cosmology, fluid dynamics and liquid crystals.

Recently, photonic counterparts of topological solitons have aroused intense interest of researchers. Optical skyrmions and optical hopfions have been experimentally demonstrated and considered to have potential applications in metrology, information encoding, and optical communication^4,5^. However, all the current researches on photonic topological solitons are restricted to spatial dimensions and lose sight of temporal or dynamic information of such structures, and hence manipulating corresponding light-matter interactions spatiotemporally remains challenging.

In the article in eLight^5^, the research group led by Prof. Qiwen Zhan from University of Shanghai for Science and Technology theoretically designed and experimentally demonstrated dynamic scalar optical hopfions weaved by nested equiphase lines in the shape of a toroidal vortex. This spatiotemporally varied optical hopfion is proposed through an analytical expression as an approximate solution to Maxwell’s equations. Numerical simulations and experimental data demonstrate that the equiphase lines are disjoint and linked closed loops in the form of links and knots with a linking number determined by the Hopf invariant. As shown in Fig. 1, each closed loop painted in one specific color corresponds to a point in the parameter space and to a circle on unit sphere in 4D space. Each closed loop is an equiphase line and all equiphase loops form complete tori that fill up the entire 3D space.

**Fig. 1:**
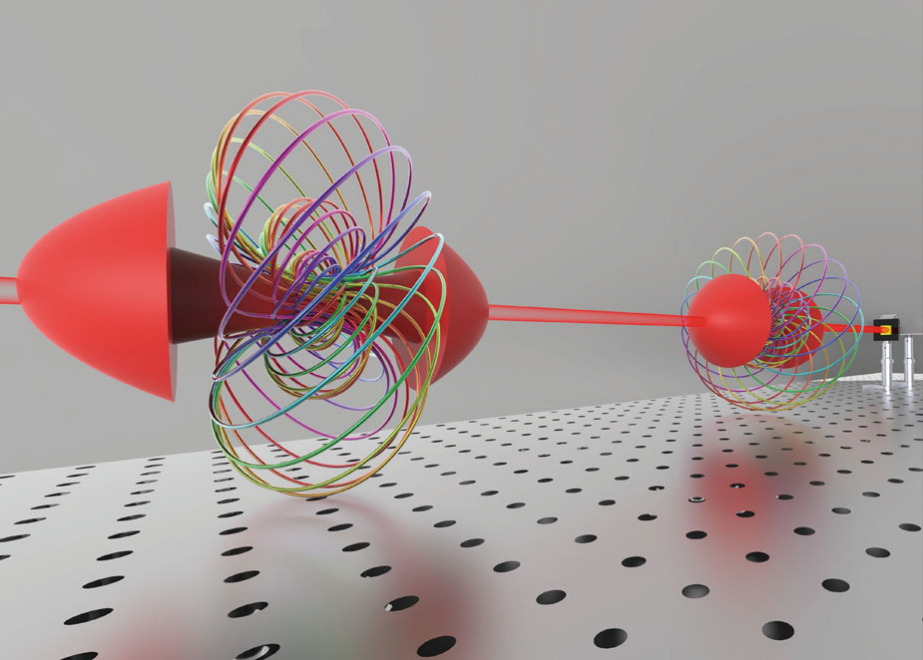
Schematic of the Hopf fibration and scalar hopfion by spatiotemporal beam shaping. The equiphase lines in a scalar hopfion present the topological features of the Hopf fibration

The key to experimentally generate such dynamic scalar optical hopfions is to manipulate poloidal and toroidal spiral phases in toroidal coordinates. In this regard, three spatial light modulators are utilized to generate spatiotemporal optical vortex and flexibly control the winding numbers of the optical hopfions. The authors perform 2D phase measurements of the poloidal phase through interfering the hopfion wave packet with a transform-limited reference pulse split from the source. The measured poloidal phase distributed in a spiral pattern in the spatiotemporal domain is well consistent with the theoretical predictions.

As the obtained dynamic scaler optical hopfions manifest as spatiotemporally structured pulses propagating in space-time, they may provide a photonic platform for studying particle-like topological states, and may find promising potential applications in light-matter interactions with spatiotemporal mode excitation, and in optical communications as high-dimensional information carriers.
